# XPR1 promotes ovarian cancer growth and regulates MHC-I through autophagy

**DOI:** 10.1016/j.gendis.2024.101507

**Published:** 2024-12-27

**Authors:** Hui Wang, Xiaodong Luo, Bo Yang, Furong Tang, Xingwei Jiang, Hongtao Zhu, Jianguo Hu

**Affiliations:** a Department of Obstetrics and Gynecology, Second Affiliated Hospital, Chongqing Medical University, Chongqing 400010, China; b Department of Obstetrics and Gynecology, Chongqing Xiushan People's Hospital, Xiushan Tujia and Miao Autonomous County, Chongqing 409900, China; c Department of Obstetrics and Gynecology, West China Second University Hospital of Sichuan University, Chengdu, Sichuan 610000, China; d Key Laboratory of Birth Defects and Related Diseases of Women and Children (Sichuan University), Ministry of Education, Chengdu, Sichuan 610000, China

**Keywords:** Autophagy, LAMP1, MHC-1, Ovarian cancer, XPR1

## Abstract

Immune checkpoint inhibitors have a poor effect in treating ovarian cancer, and the specific mechanism is unknown. The purpose of this research was to investigate the impact of XPR1 on controlling autophagy in ovarian cancer. The findings suggested an increase in XPR1 expression in ovarian cancer tissues. The elevated level of its expression was linked to the stage of ovarian cancer, as well as overall survival and progression-free survival. XPR1 enhanced the growth and spread of ovarian cancer while suppressing autophagy. Moreover, XPR1 suppressed autophagy flux by interacting with LAMP1 and the PI3K/Akt/mTOR pathway. XPR1 controlled the positioning and production of MHC-I molecules on the surfaces of ovarian cancer cells via autophagy. Silencing XPR1 combined with the autophagy inhibitor chloroquine significantly inhibited tumor growth in mouse ovarian cancer models. In conclusion, the findings indicate that XPR1 could serve as a promising target for the diagnosis and treatment of ovarian cancer. Combined autophagy inhibitors may improve the sensitivity of ovarian cancer immunotherapy.

## Introduction

Evading anti-tumor immune response is an important survival strategy for various tumors. Programmed death-1 (PD-1) and cytotoxic T lymphocyte-associated protein-4 (CTLA-4), two proteins present on the surface of cytotoxic T cells (also referred to as CD8^+^ T cells), can inhibit immune reactions. Two antibodies, each targeting these two inhibitors of protein, are widely used to drive anti-tumor immune responses. Therefore, the emergence of PD-1 and CTLA-4 antibodies opens a new avenue for treating solid tumors.^1^^,^^2^ However, it is less effective in solid tumors such as pancreatic and ovarian cancers. CD8^+^ T cells' ability to inhibit tumor growth relies on their ability to identify and attach to cancer cells by recognizing the major histocompatibility complex (MHC-I class molecule peptide fragments) on their surface. New findings indicate that the MHC-I antigen is present on the normal ductal epithelial cell membrane in the pancreas and functions as an antigen presenter. However, MHC-I protein is degraded by autophagosome transport to autophagolysosomes in pancreatic ductal adenocarcinoma. Consequently, the MHC-I protein expression is decreased in pancreatic ductal adenocarcinoma. The down-regulation of MHC-I protein is mediated by selective autophagy degradation.^3^ Hydroxychloroquine, an autophagy inhibitor, or the deletion of autophagy-related genes ATG13 and ATG14 can prevent the breakdown of MHC-I protein and enhance the presence of CD8^+^ T cells in tumor areas, leading to an immune response against tumors. In breast and colon cancers, the sensitivity of tumor cells to T-cell killing was significantly enhanced by knocking out autophagy-related genes RB1CC1 (RB1 inducible coiled-coil 1), ATG9A, and ATG12. The anti-cancer effect of PD-1 and CTLA-4 checkpoint inhibitors was enhanced.^4^ The knockout of the autophagy-related gene ATG12 makes tumor cells more sensitive to T-cell killing in breast, colorectal, and kidney cancers and melanoma.^5^ The absence of MHC-I antigens in the ID8 ovarian cancer cell line indicates a connection between immune evasion in ovarian cancer and MHC-I. Ovarian cancer cell lines OVCAR8 and CAOV3 exhibit reduced levels of MHC-I antigen expression, leading to resistance against T-cell cytotoxicity.^6^ However, the mechanism of low expression of MHC-I in epithelial ovarian cancer remains unclear.

Ovarian cancer is a malignant tumor with the worst prognosis among gynecological tumors. The high mortality rate is mainly related to tumor metastasis. Epithelial ovarian cancer is the main pathological type of ovarian tumor. Although there have been improvements in surgical techniques, chemotherapy, and follow-up care, the 5-year survival rate for epithelial ovarian cancer remains approximately 50%. Early-stage ovarian cancer has occulted onset and no specific signs and lacks specific and sensitive diagnostic markers. Therefore, early diagnosis is difficult. About 70% of patients with ovarian cancer have already developed abdominal or distant metastases when first diagnosed.^7^, ^8^, ^9^ Immunotherapy has made remarkable progress in solid tumors in recent years. Nevertheless, less than 15% of patients with epithelial ovarian cancer experience positive outcomes from immune checkpoint inhibitor treatment, including PD-1 and CTLA-4 antibodies. Therefore, exploring the immune escape mechanism of ovarian cancer is of great importance for immunotherapy.^10^

Autophagy is the process in which autophagosomes are created from the bilayer membrane detached from the ribosome-free area of the rough endoplasmic reticulum. These autophagosomes enclose a portion of the cytoplasm along with degraded organelles, proteins, and other elements before merging with lysosomes. Also, a series of proteases degrade the contents of the autophagosomes, and the autophagy flow is completed.^11^, ^12^, ^13^ The enclosed materials break down to meet the cell's metabolic requirements and to renew specific organelles. At present, lysosomal-associated membrane protein 1 (LAMP1) is utilized as an indicator of lysosomes to identify the creation of autophagolysosomes. Furthermore, LAMP1 plays a role in controlling autophagy in cancer cells as well.^14^^,^^15^ Autophagy is a well-preserved process within cells that safeguards against harmful triggers and sustains cellular balance by breaking damaged proteins and organelles. Autophagy is at a low level in normal cells. However, it is activated under the effect of metabolism, chemotherapeutic drugs, hypoxia, and other conditions as well as the changes in the microenvironment, thus maintaining the stability of cells. Moderate levels of autophagy support the viability of cancer cells in challenging conditions, such as exposure to radiation or chemotherapy and limited nutrient availability. Whether autophagy plays a role in promoting or suppressing cancer is still debated. It can either promote or inhibit the growth of malignant tumors. This suggests that autophagy may play different roles in different stages of tumorigenesis.^16^, ^17^, ^18^ Nevertheless, the function and process of autophagy in the immune response to ovarian cancer remain uncertain.

Discovered in 1999, xenotropic and polytropic retrovirus receptor 1 (XPR1) was recognized as a receptor on the cell surface that specifically binds to xenotropic and polytropic murine leukemia virus. This protein consists of 696 amino acids and has several transmembrane regions.^19^ XPR1 plays a crucial role in the recruitment of G proteins and is necessary for signal transduction in G protein-coupled receptors. XPR1 is involved in tumor cell growth, metastasis, and invasion. In human cells, XPR1 is involved in the transmembrane transport of inorganic salts, thereby regulating cellular phosphate homeostasis.^20^ Activation of the NF-κB signaling pathway by XPR1 can enhance the aggressive biological development of tongue squamous cell carcinoma. XPR1 facilitated the growth, invasion, and metastasis of ovarian cancer cells in epithelial ovarian cancer with the CRISPR/Cas9 system. In addition, there was an increase in the number of XPR1 copies and mRNA expression. In ovarian cancer cells, either knocking out XPR1 or inhibiting XPR1 with drugs results in the harmful buildup of phosphate inside the cells, ultimately causing cell death. Nevertheless, the function and operation of the XPR1 protein in autophagy and evasion of the immune system by ovarian cancer cells remain unknown.

The primary objective of this research was to examine the involvement and impact of XPR1 in ovarian cancer, with a specific emphasis on its function and process in autophagy. Furthermore, the research aimed to explore the impact of XPR1 on MHC-I regulation. This study found that XPR1 suppressed autophagy flux by interacting with LAMP1 and the PI3K/Akt/mTOR pathway.

## Materials and methods

### Tissue specimens

The ovarian cancer tissue chip was purchased from Shanghai WEIAO Biotechnology Co., Ltd. (product number: ZL-OVA961; WEIAOBIO, Shanghai, China). This microarray included 88 ovarian cancer tissues and 8 normal ovarian tissues. Concurrently, healthy ovarian tissues were obtained from individuals who had total hysterectomy and bilateral salpingo-oophorectomy for noncancerous gynecological conditions at the Gynecology Department of the Second Affiliated Hospital of Chongqing Medical University. The study documents and use of archived cancer tissues were approved by the ethics committee at Chongqing Medical University (approval No. (2023)123). In this study, all participants gave informed consent and signed the consent form.

### Genome-wide screening

SKOV3 cells expressing GFP-LC3-RFP and Cas9 were infected with the pooled lentiviral library (multiplicity of infection: 0.5–0.7) and selected with puromycin. Two weeks after infection, cells were starved for 24 h and subjected to fluorescence-activated cell sorting (FACS) using MoFlo AstriosEQ (Beckman Coulter). After sorting, cells were cultured, expanded, and subjected to FACS again. The enrichment by FACS was performed two times. Besides, DNA was extracted from cells in the control group and experimental group, the coding sequence of single-guide RNA (sgRNA) was amplified by PCR, and the sgRNA library was sequenced by high-throughput sequencing.

### Next-generation sequencing

48 h after SKOV3 cells were transfected with XPR1 siRNA, the cells were collected and total RNA was extracted. The total RNA of SKOV3 was extracted by RNeasy Plus Micro Kit (Qiagen,74,134) according to the manufacturer's instructions. RNA quality was checked using 1% agarose gel electrophoresis and RNA Nano 6000 Assay Kit (Agilent, 5067–1511). Shanghai Lifegenes Technology Co., Ltd. participated in library construction, sequencing, and data analysis. Gene ontology (GO) enrichment analysis of differentially expressed genes was implemented using the clusterProfiler R package (v3.12.0). GO terms with a *P*-value less than 0.05 were considered significantly enriched by differentially expressed genes.

### Cell cultivation, transfection method, and chemicals

A2780, SKOV3, and ID8 cells derived from ovarian cancer were grown in RPMI 1640 medium (Sigma–Aldrich, R8758) supplemented with 10% fetal bovine serum (Gibco, C11995500BT) and antibiotics. The cells were cultured at 37 °C in an environment with 5% carbon dioxide. Genepharma (Genepharma, Shanghai, China) synthesized the siRNAs. The order of transfection events was the following: XPR1-975: 5′-GGCCCUUGAUAAGAAUCUATT-3'; XPR1-1860: 5′-GUGCCAUAAUAGAGGAUGUTT-3'; XPR1-1383: 5′-GCCUGUCAGUGAUACUGAUTT-3'; GANAB-109: 5′-GUGGAUAGAAGCAACUUUATT-3'; GANAB-845: 5′-GCCUCUACAAUUUGGAUGUTT-3'; GANAB-1584: 5′-GUUCAGCUAUGACAAUUAUTT-3'; ILVBL-852: 5′-CCUGGUAUUUAGAGAAUUATT-3'; ILVBL-1239: 5′-GCAGCAAGAUCAUCAUCGUTT-3'; ILVBL-1509: 5′-CGCUACCUGACAACUCAAUTT-3'; ITGB4-799: 5′-GUGGAUGAGUUCCGGAAUATT-3'; ITGB4-5002: 5′-GGUGUCAUCACCAUUGAAUTT-3'; ITGB4-3581: 5′-CCAGGAAGAUCCAUUUCAATT-3'; Negative control: 5′-UUCUCCGAACGUGUCACGUTT-3'; LAMP1: 5′-UGCUGCCUUCUCAGUGAACUA-3'; XPR1-Mus-1005: 5′-GGCCCUUGAUAAGAAUCUATT-3'; XPR1-Mus-1108: 5′-CCUCAUCUUUGAACUUAAUTT-3'; XPR1-Mus-1810: 5′-GGGUCUCUUUGAUAAGAAUTT-3'; XPR1-Mus-1549: 5′-GCGAGCCAUUGUUCAAUGUTT-3'. Genepharma (Genepharma, Shanghai, China) supplied lentiviral vectors containing shRNA targeting XPR1, as well as the XPR1-lentiviral expression vector (GeneCopoeia, T0271, Guangzhou, China). After the cells were cultured in a six-well plate until the confluence reached about 70%, siRNA and transfection reagent (G04009, Genepharma, Shanghai, China) were mixed according to the instructions and added to the culture medium. 48 h after transfection, the cells were collected for PCR or Western blot detection.

### Immunohistochemistry analysis

Immunohistochemistry kits were purchased from Beijing Zhong Shan-Golden Bridge Biological Technology Co., Ltd. (SAP-9100, ZSGB-BIO, China). Immunohistochemistry experimental procedures were performed according to the manufacturer's instructions. Tissue microarrays were heated at 60 °C for 1 h, treated with xylene to remove paraffin, gradually hydrated with alcohol, and then placed in a microwave oven with citrate for antigen retrieval. Endogenous peroxidase activity was eliminated by incubation with 3% hydrogen peroxide in deionized water for 15 min. Animal serum lacking immunity was placed and left to incubate at ambient temperature for 15 min. The primary antibody was added slowly and left to incubate at 4 °C overnight in a 1:200 dilution. Following the wash the next day, secondary antibodies were slowly added and incubated at 37 °C for 30 min. The cells were incubated with streptomycin-avidin-peroxidase for 15 min. Color development was achieved using DAB for 3 min, and then counterstained with hematoxylin, dehydrated, and sealed with gum. The pictures were examined and measured using Image-Pro Plus 6.0 software.

### Co-immunoprecipitation test

The co-immunoprecipitation samples were prepared as follows: cells were harvested and lysed in immunoprecipitation buffer containing 1% Triton X-100, Complete Mini protease inhibitor cocktail (Roche Applied Sciences, Basel, Switzerland), and 1 M phosphate-buffered saline (pH 7.4) on ice for 30 min. The cell lysate was centrifuged at maximum speed at 4 °C for 30 min to obtain the supernatant. The soluble fraction was incubated with an anti-XPR1 antibody on a rotator at 4 °C overnight. After washing three times with tris buffered saline with Tween 20, Dynabeads Protein A (Invitrogen AG, Switzerland) was added, followed by incubation with rotation at room temperature for 2 h. The protein complex was eluted by boiling with a loading buffer. Afterward, western blotting was used to detect LAMP1 expression.

### Quantitative reverse transcription PCR

Total RNA was extracted following the instructions for TRIzol, and then the extracted RNA was reverse transcribed following the reverse transcription kit instructions (Bio-Rad Laboratories, 4,106,228). The expression level of XPR1 mRNA was measured using cDNA as a template. An internal control was employed in the form of GAPDH. GeneCopoeia supplied the primers (product No. HQP116040, GeneCopoeia, China). The parameters for real-time PCR were set at 95 °C for 30 s, followed by 40 cycles of 95 °C for 5 s and 60 °C for 30 s, and then a final step of 95 °C for 10 s, 65 °C for 5 s, and 95 °C for 5 s. Target gene results were expressed as 2^−ΔΔCt^.

### Western blotting

Protein extraction and determination of protein concentration were performed following the instructions of the protein extraction kit (Beyotime Biotechnology, P0033) and BCA kit, respectively. The separation gel and the concentration gel were prepared following the reagent ratio instructions, and the protein was loaded and electrophoresed in an appropriate proportion. Next, the protein was moved to the PVDF membrane, where it was blocked using 5% skim milk and then exposed to the primary antibody (diluted 1:1000) at 4 °C overnight. The following day, the membrane was washed with tris buffered saline with Tween 20 and then exposed to the secondary antibody at room temperature for 1 h. Then, the membrane was washed and processed with an enhanced chemiluminescence kit and photographed. An internal control was employed in the form of GAPDH.

The primary antibodies used in the study included polyclonal rabbit anti-XPR1 (Abcam, ab97483); rabbit polyclonal antibody to LAMP1 (Abcam, ab24170); rabbit polyclonal antibody to PI3K (Abcam, ab154598); rabbit polyclonal antibody to p-PI3K (CST, 17366); rabbit polyclonal antibody to p-Akt (CST, 4060); rabbit polyclonal antibody to Akt (CST, 9272); rabbit polyclonal antibody to mTOR (CST, 2972); rabbit polyclonal antibody to p-mTOR (CST, 2971); rabbit polyclonal antibody to ULK1 (CST, 8054); rabbit polyclonal antibody to p-ULK1 (Abcam, ab203207); and rabbit polyclonal antibody to GAPDH (CST, 2118). Band density was assessed by a gel imaging system and then compared with an internal standard.

### Immunohistochemical staining

The cells were treated with a stabilizing agent for 10 min, obstructed with a solution for 1 h, and softly agitated with a mixer. Antibodies targeting XPR1 (Abcam, ab150586) and LAMP1 (Abcam, ab25630) were applied, and then incubated with goat anti-rabbit IgG (FITC) (Abcam, ab25630) at 37 °C for 60 min and goat anti-mouse IgG (TRITC) (Abcam, ab6786) in the dark for 60 min. DAPI was used to stain the nuclei for 10 min. Subsequently, the images were observed by laser confocal microscopy. The results were quantified using the ImageJ software. The experiments were conducted thrice for accuracy.

### Staining of lysosomes

LysoTracker Red was purchased from Beyotime (product number: C1046). The concise process involved adding a small quantity of LysoTracker Red to the cell culture solution in a 1:20,000 ratio, resulting in a final concentration of 50 nM. The cells were preheated at 37 °C for 10 min. After removing the cell culture medium, the cells were exposed to a LysoTracker Red staining solution that had been prepared and preincubated at 37 °C for 60 min. After discarding the LysoTracker Red-stained working solution, a new cell culture medium was introduced. Subsequently, the images were observed by laser confocal microscopy. The results were quantified using the ImageJ software. The experiments were conducted thrice for accuracy.

### Concurrent fluorescence of mRFP-GFP-LC3

A tandem monomeric RFP-GFP-tagged LC3 lentiviral vector was used to monitor autophagy flux as in our previous study.^21^ SKOV3 and A2780 cell lines were infected with mRFP-GFP-LC3 for 48 h, followed by treatment with puromycin (5 μg/mL) and subsequent culturing in the 5 μg/mL rapamycin medium. They were transfected with XPR1 and control siRNA and then cultured for another 48 h. Confocal laser images were taken and analyzed.

Autophagy inducer Torin1 (10 μM) was added 24 h after transfection with XPR1 overexpression lentiviral vector or control vector in the mRFP-GFP-LC3 infected SKOV3 and A2780 cells. Confocal laser images were taken and analyzed. The number of LC3 dots was measured with the assistance of ImageJ software. The experiments were conducted thrice for accuracy.

### EdU proliferation assay

Cell proliferation was identified using a Cell-Light EdU imaging detection kit from Ruibo Biotechnology in Guangzhou, China, in accordance with the provided guidelines.

### Matrigel invasion assay

In transwell invasion experiments, the Matrigel basement membrane matrix (BD Biosciences, MA, USA) was initially applied to the top of a 6.5-mm polycarbonate transwell filter membrane (8-μm pore size; Corning, NY, USA). After pre-treatment at 37 °C for 2 h, the top chamber was filled with 5 × 10^4^ ovarian cancer cells and left to incubate for 48 h. The cells that migrated to the bottom of the chamber were then fixed using 4% paraformaldehyde, stained with 0.5% crystal violet (Beyotime), and counted using a microscope. Five fields for each transwell chamber were assessed. Every individual field was examined and captured under 200 × magnification.

### Ovarian cancer mouse model

Twenty female nude mice aged 6–8 weeks were purchased from the Experimental Animal Center of Chongqing Medical University and divided into 4 groups, with 5 mice in each group. Animal experiments followed the procedures approved by the Committee on the Use and Care of Animals at Chongqing Medical University in China, adhering to the institution's guidelines. SKOV3 cells were implanted subcutaneously into the left armpit (5 × 10^6^ cells in 200 uL per mouse) after being transfected with lentiviral vectors XPR1-975 shRNA (referred to as KD) and control (referred to as NC-KD), as well as XPR1-overexpressing lentiviral vector (referred to as OE) and control lentiviral vector (referred to as NC-OE). After 28 days, the mice were euthanized with ether anesthesia. The size and weight of the tumors were measured. Afterward, the tumor tissues were fixed with 4% paraformaldehyde, and protein expression was detected by immunohistochemistry.

### ID8 cells were used for tumor formation in C57 mice

The cells were divided into four groups: XPR1-1108 shRNA (named KD) group, control (named NC-KD) group, XPR1-1108 shRNA plus chloroquine group, and control plus chloroquine group. One week after the subcutaneous injection of tumor cells, chloroquine (80 mg/kg) was administered intraperitoneally to the cells in the XPR1-1108 shRNA plus chloroquine group and the control plus chloroquine group. Chloroquine was injected subcutaneously once daily for 3 weeks, and then the animals were sacrificed. After the sacrifice, the mean volume was calculated on day 28 after the ID8 cell injection. At the same time, immunohistochemical detection of related protein expression was conducted for part of each tumor tissue.

### Statistical analysis

SPSS 17.0 was utilized for all statistical analyses. Every test was conducted three times. Statistical analysis was performed by student's *t*-test or analysis of variance (ANOVA). The chi-square test was employed to assess the relationships between increased XPR1 expression and the clinicopathological characteristics of ovarian cancer specimens. The information was displayed as average plus/minus deviation. A *P*-value less than 0.05 was used to define statistical significance.

## Results

### CRISPR-Cas9 library identified XPR1 as a potential gene regulating autophagy

**Figure 1:**
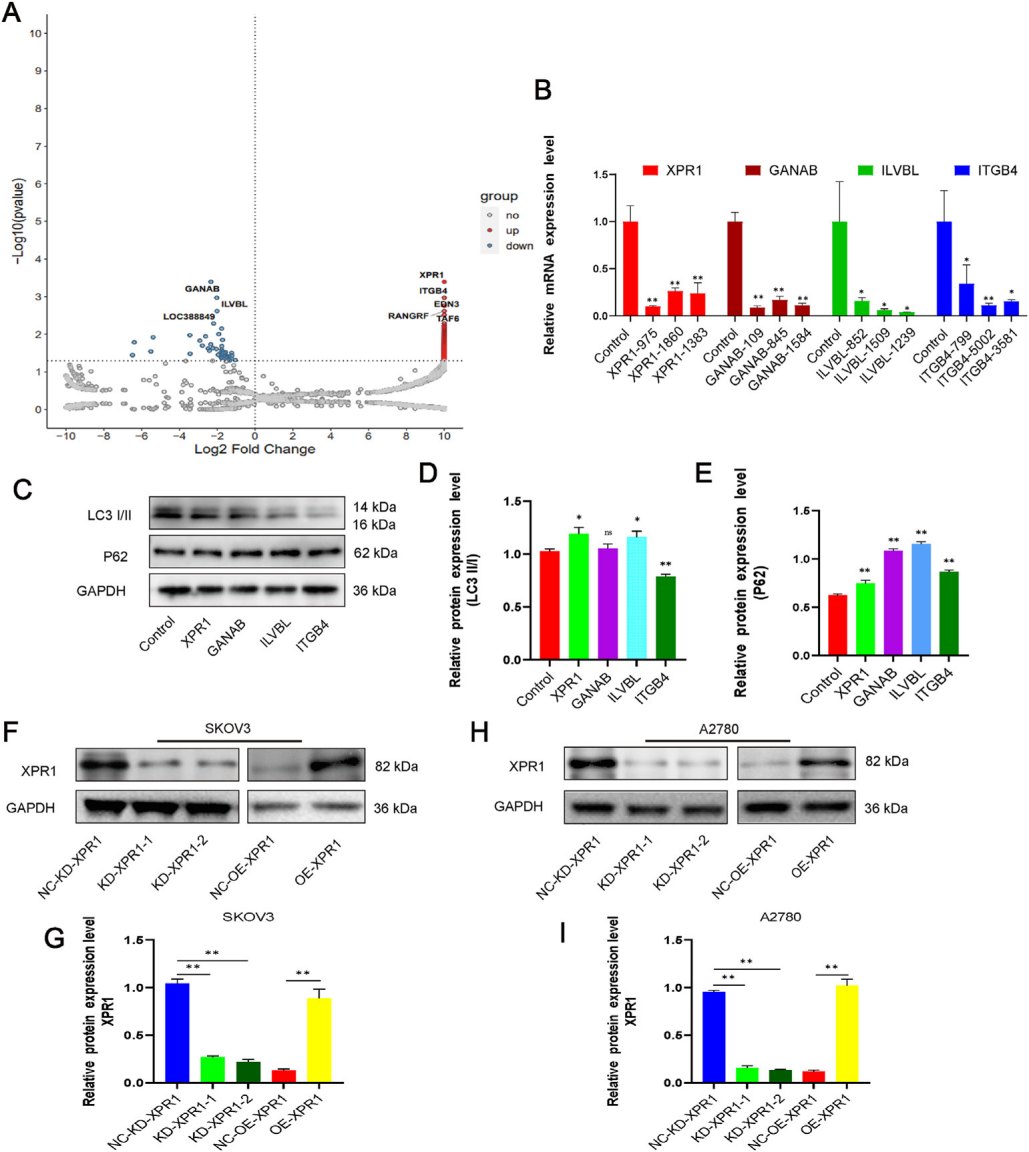
CRISPR-Cas9 library identified XPR1 as a potential gene regulating autophagy. SKOV3 cells expressing GFP-LC3-RFP and Cas9 were infected with the pooled lentiviral library and selected with puromycin. After 2 weeks, the cells were starved for 24 h. Flow cytometry was used to sort cells with increased or decreased RFP/GFP ratio greater than 2. The cells were cultured, expanded, and screened by flow cytometry. Three screenings were performed by flow cytometry. The cells were then sequenced. **(A)** Volcano map of selected genes potentially regulating autophagy. **(B)** SiRNAs corresponding to XPR1, GANAB, ILVBL, and ITGB4 and control siRNA were transfected into SKOV3 cells. The RNA was extracted 48 h after transfection. Then, the mRNA expression level was detected by quantitative PCR. **(C)** SKOV3 cells were transfected with XPR1-975 siRNA, GANAB-109 siRNA, ILVBL-1239 siRNA, and ITGB4-5002 siRNA and control siRNA. The protein was collected 48 h after transfection, and then the expression of LC3-II and P62 was detected by Western blot. **(D)** XPR1-975 siRNA (named XPR1-1), XPR1-1860 siRNA (named XPR1-2), and control siRNA (named NC-KD-XPR1) were transfected into SKOV3 and A2780 cells. XPR1-overexpressing lentiviral vectors (named OE-XPR1) or control lentiviral vectors (named NC-OE-XPR1) were transfected into SKOV3 and A2780 cells. The protein was collected and the expression level of XPR1 protein was detected by Western blot 48 h after transfection. Error bars represent standard deviation. ∗∗*P* < 0.01.

We screened a genome-wide CRISPR-Cas9 library in SKOV3 cell lines to identify the genes potentially involved in autophagy. The ovarian cancer SKOV3 cell line was transfected with the autophagy double-labeled lentivirus vector (mRFP-GFP-LC3), and then the autophagy model of SKOV3 cells was constructed by nutrient deprivation. Flow cytometry was used to screen cells with increased or decreased RFP/GFP ratio greater than 2 for further culture (Figs. S1A and B). Then, the cells were directly applied to sgRNA sequencing. We screened 121 genes with an increased mRFP/GFP ratio. A total of 101 genes had a reduced mRFP/GFP ratio (Fig. 1A). The genes mTOR, AMPK, ULK1, ATG7, and ATG5 were screened out. Because the role of these molecules in autophagy has been confirmed, we chose some of the remaining molecules to study. We selected four genes with higher scores for further study. These four genes encode proteins that belong to ion channels or receptors and may be used in clinical practice. Initially, we employed quantitative PCR to confirm the effectiveness of siRNA in suppressing XPR1, GANAB, ILVBL, and ITGB4 gene expression. The siRNA targeted by these four genes could significantly reduce the mRNA expression of the corresponding genes (Fig. 1B). Among these four genes, LC3-II/LC3-I increased most significantly and P62 decreased most significantly after XPR1 silencing (Fig. 1C–E). Therefore, we chose XPR1 for further study. XPR1 was either silenced or overexpressed in the ovarian cancer cell lines SKOV3 and A2780. Following XPR1 silencing, the XPR1 protein expression level notably decreased, while it significantly increased with XPR1 overexpression (Fig. 1F–I).

### Aberrant expression of XPR1 in ovarian carcinoma

**Figure 2:**
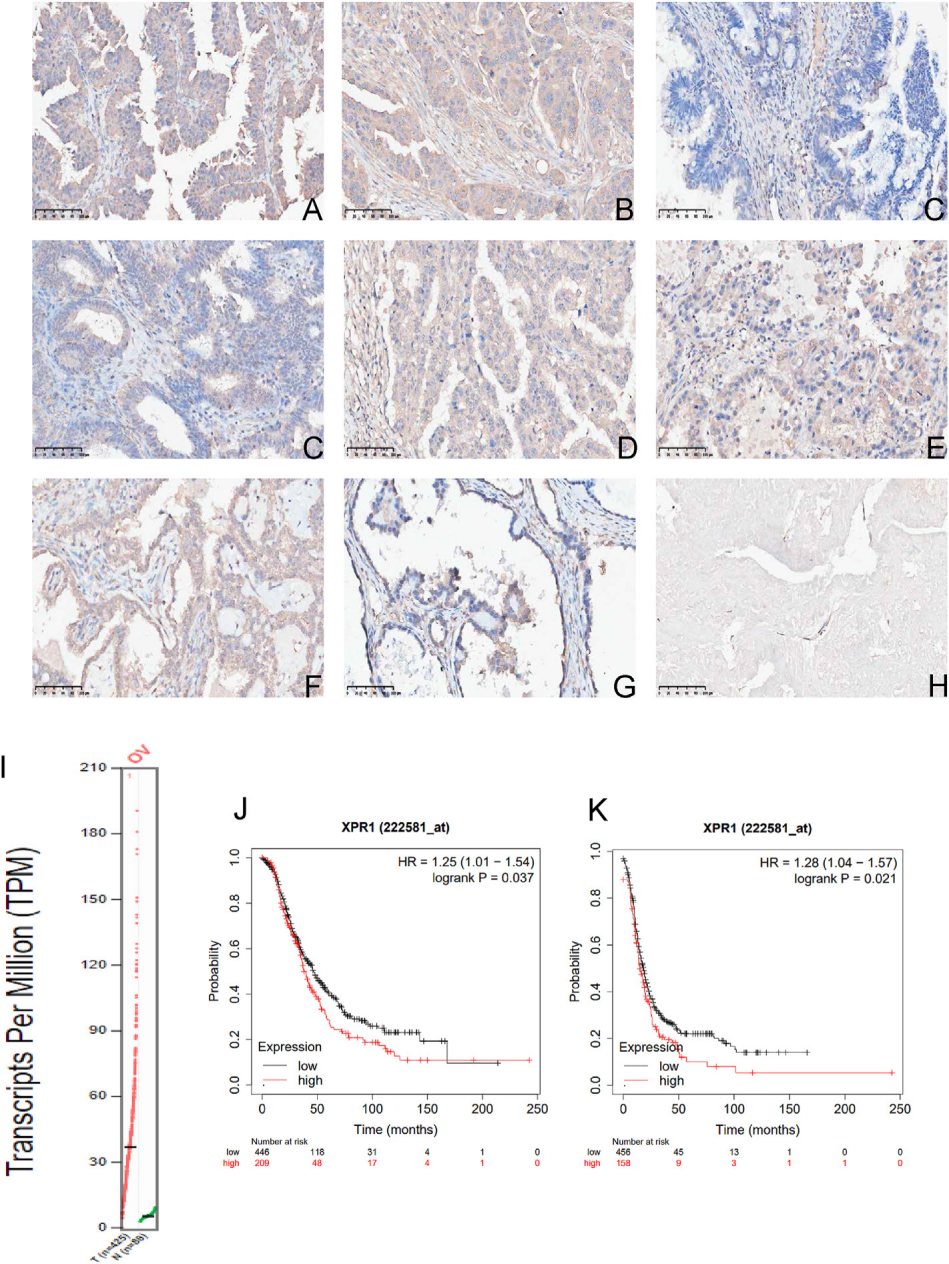
Aberrant expression of XPR1 in ovarian carcinoma. The expression pattern of XPR1 in ovarian cancer was detected by immunohistochemistry. **(A, B)** High-grade serous ovarian cancer at stage III. **(C)** High-grade serous ovarian cancer at stage I. **(D)** Endometrioid carcinoma at stage I. **(E)** Ovarian mucinous carcinoma at stage III. **(F)** Ovarian mucinous carcinoma at stage I. **(G)** Ovarian clear cell carcinoma at stage III. **(H)** Ovarian clear cell carcinoma at stage I. **(I)** Normal endometrial tissue. **(J)** Expression of XPR1 in ovarian cancer and normal ovarian tissue was analyzed using the TCGA database. **(K)** Overall survival curve of XPR1 in ovarian cancer by analyzing TCGA database. **(L)** Progression-free survival curve of XPR1 in ovarian cancer by analyzing TCGA database. Scale bar: 100 μm.

We first examined the expression of XPR1 to clarify its role in ovarian cancer. We used immunohistochemistry to detect the expression pattern of XPR1 in ovarian cancer. XPR1 was found to be mainly localized in the cell and membrane in ovarian cancer tissues. It was moderately or strongly expressed in ovarian cancer but not in normal ovarian tissue (Fig. 2A–I). We found that the higher the expression of XPR1 in ovarian cancer tissue, the higher the clinical stage of ovarian cancer (Fig. 2A–I and Table 1). Simultaneously, an examination of the TCGA database revealed an elevation in XPR1 protein levels in ovarian cancer in contrast to normal ovarian tissue. Moreover, XPR1 expression was associated with ovarian cancer's overall survival and progression-free survival. The higher the XPR1 expression, the worse the prognosis (Fig. 2J–L).

**Table 1:** Clinicopathological characteristics and their associations with the expression lecel of XPR1 in ovarin cancer tissue.

Clinicopathological variable	Case no 88	XPR1 level		χ^2^ value	*P* value
		High no.31(%)	low no. 57(%)		
Age at diagnosis, year				3.228	0.072
＜60	59	17(28.81%)	42(71.19%)		
≥60	29	14(48.28%)	15(51.72%)		
FIGO stage				7.014	0.008
I/II	48	11(22.92%)	37(77.02%)		
III/IV	40	20(50.00%)	20(50.00%)		
Metastases of the tumor				8.162	0.004
YES	26	15(57.69%)	11(42.31%)		
NO	62	16(25.81%)	46(74.19%)		

### XPR1 suppressed the flow of autophagy in SKOV3 and A2780 ovarian cancer cell lines

**Figure 3:**
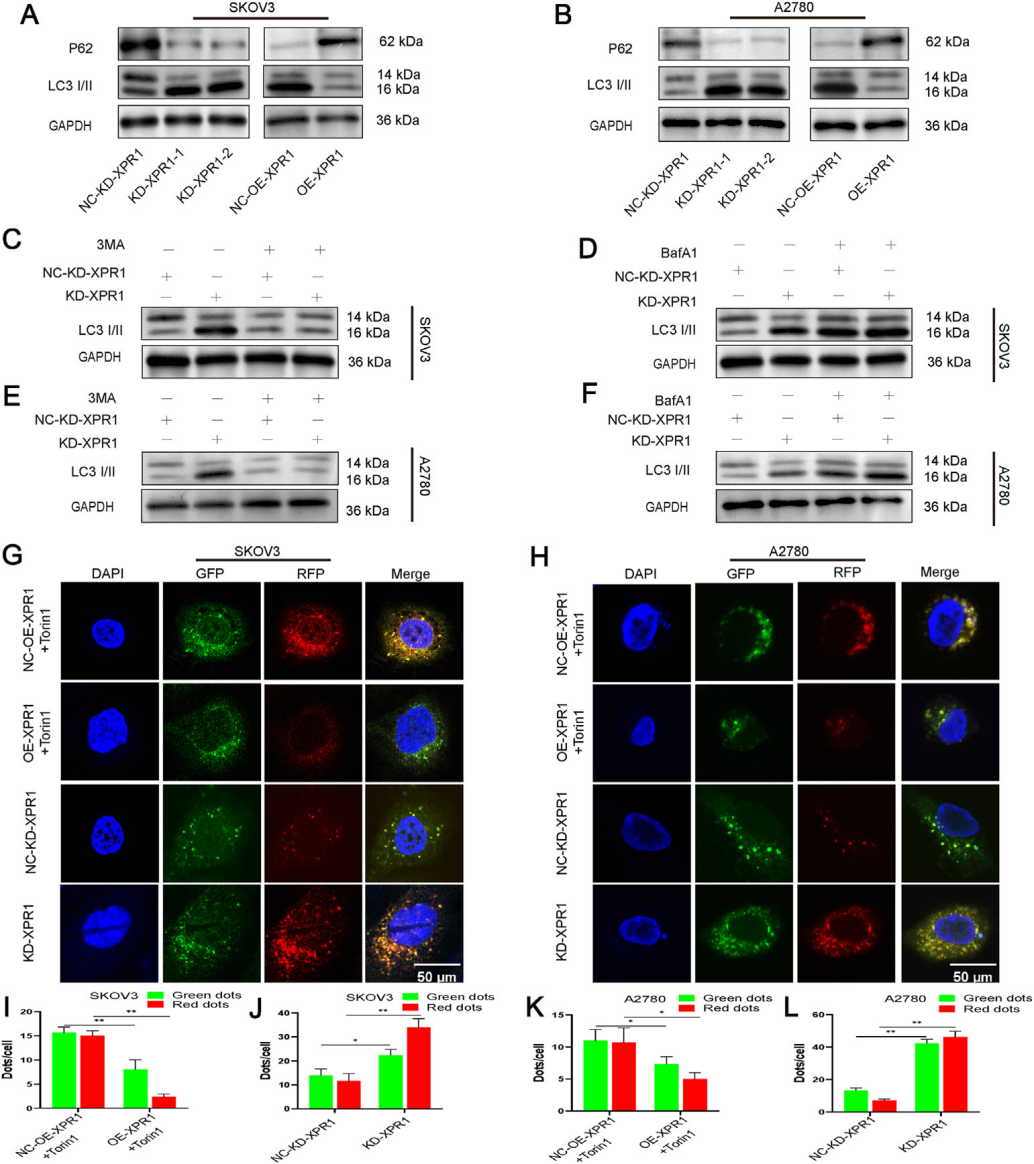
XPR1 inhibited autophagy flux in ovarian cancer cell lines SKOV3 and A2780. **(A)** XPR1-975 siRNA (named XPR1-1), XPR1-1860 siRNA (named XPR1-2), and control siRNA (named NC-KD-XPR1) were transfected into SKOV3 and A2780 cells. XPR1-overexpressing lentiviral vectors (named OE-XPR1) or control lentiviral vectors (named NC-OE-XPR1) were transfected into SKOV3 and A2780 cells. The protein was collected and the expression level of P62 and LC3-II protein was detected by Western blot 48 h after transfection. **(B, C)** XPR1-975 siRNA (named KD-XPR1) and control siRNA (named NC-KD-XPR1) were transfected into SKOV3 and A2780 cells. 3-MA or BAFA1 was added 48 h after transfection, and the cell protein was collected and the expression of LC3-II was detected by Western blot after another 48 h. **(D)** SKOV3 and A2780 cells were transfected with KD-XPR1, NC-KD-XPR1, OE-XPR1 plus Torin1, and NC-OE-XPR1 plus Torin1. mRFP-GPF-LC3 was then transfected, and the number of red and green dots was detected by laser confocal detection 48 h after transfection. ImageJ was then used for the statistical analysis of green and red dots. Original magnification: 200 × . Scale bar: 50 μm. Each experiment was repeated three times. Error bars represent standard deviation. ∗*P* < 0.05, ∗∗*P* < 0.01.

The impact of XPR1 on autophagy flux was investigated by analyzing the levels of LC3-II and P62 in SKOV3 and A2780 cell lines following XPR1 silencing or overexpression. After silencing XPR1, we observed an increase in the ratio of LC3-II to LC3-I and a decrease in the expression level of P62 protein. Following XPR1 overexpression, there was a reduction in the LC3-II/LC3-I ratio and an increase in the level of P62 protein (Fig. 3A, B; Figs. S1C–F). We added 3-MA and BafA1 to determine whether XPR1 was involved in the autophagosomal or autophagolysosomal phase. The ratio of LC3-II/LC3-I was observed to rise following XPR1 suppression, with this rise being notably suppressed upon the introduction of 3-MA. This increase was partially suppressed by adding BafA1. The findings suggested that XPR1 controlled autophagy flow primarily during the beginning phase of autophagy and to some extent during the lysosomal phase (Fig. 3C–F; Fig. S1G and H). Autophagy flux was detected using the lentiviral vector mRFP-GFP-LC3. Silencing XPR1 in SKOV3 and A2780 ovarian cancer cells resulted in a notable rise in red dots. Upon introducing Torin1 into SKOV3 and A2780 cells to trigger autophagy, the up-regulation of the XPR1 gene resulted in a reduction in red dots and an increase in green dots (Fig. 3G–L; Fig. S1I and J). These results indicated that XPR1 inhibited autophagy flux and enhanced the growth and spread of ovarian cancer cells.

**Figure 4:**
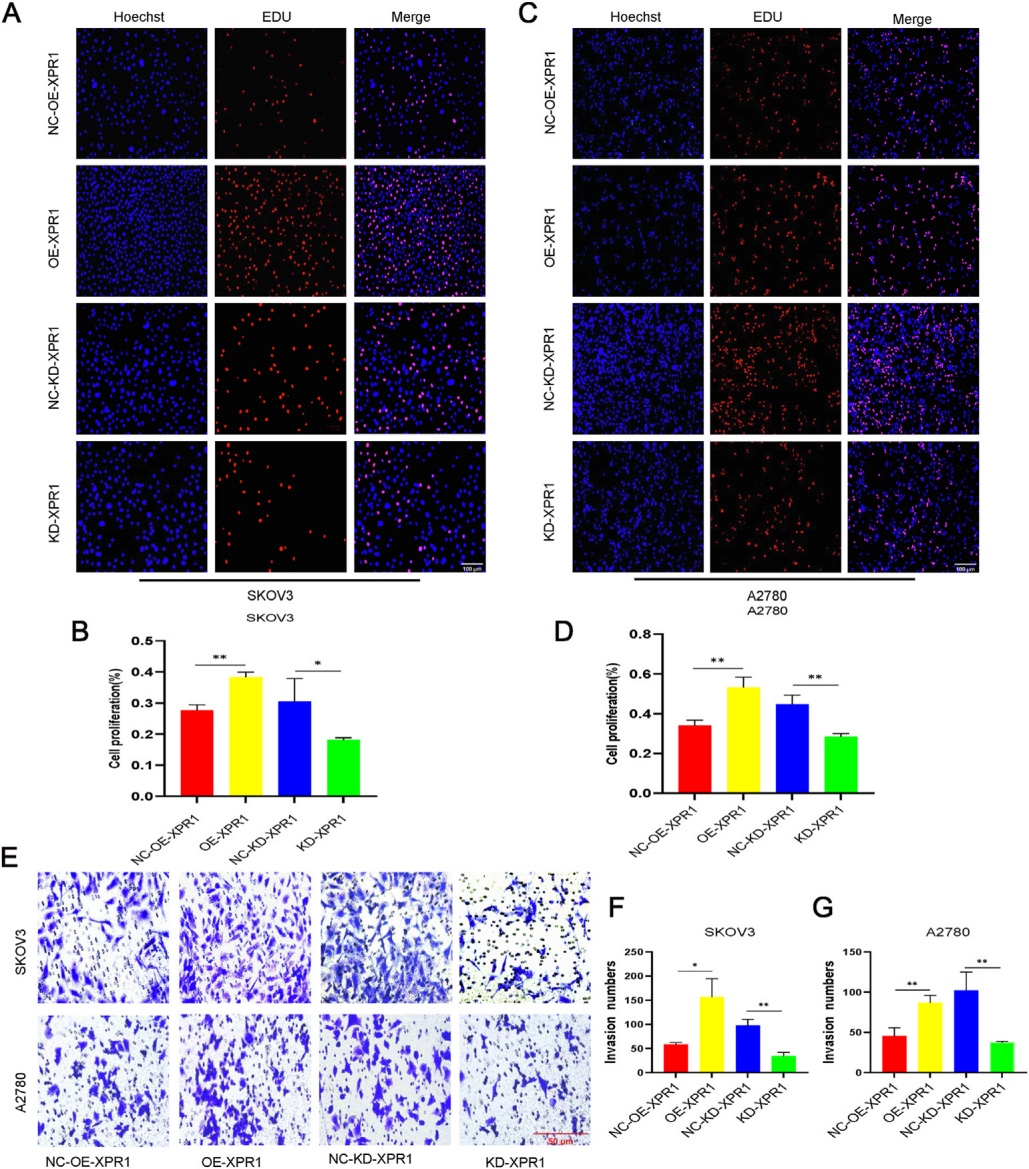
XPR1 promoted the proliferation and metastasis of ovarian cancer cells. **(A)** SKOV3 and A2780 cells were transfected with KD-XPR1, NC-KD-XPR1, OE-XPR1, and NC-OE-XPR1. The cell proliferation was measured by the EdU assay 48 h after transfection. Original magnification: 200 × . Scale bar: 100 μm. **(B)** SKOV3 and A2780 cells were transfected with KD-XPR1, NC-KD-XPR1, OE-XPR1, and NC-OE-XPR1. The transwell cell migration assay was used to detect the cell migration ability. Original magnification: 200 × . Scale bar: 50 μm. Each experiment was repeated three times. Error bars represent standard deviation. ∗*P* < 0.05, ∗∗*P* < 0.01.

We examined the effect of silencing or overexpressing XPR1 on SKOV3 and A2780 ovarian cancer cells to clarify the effect of XPR1 on the malignant biology of ovarian cancer. SKOV3 and A2780 cells were found to have reduced proliferation and metastasis capacity after silencing XPR1. Overexpression of XPR1 led to increased proliferation and metastasis of SKOV3 and A2780 cells (Fig. 4A–G).

### XPR1 interacted with LAMP1 and regulated its expression

**Figure 5:**
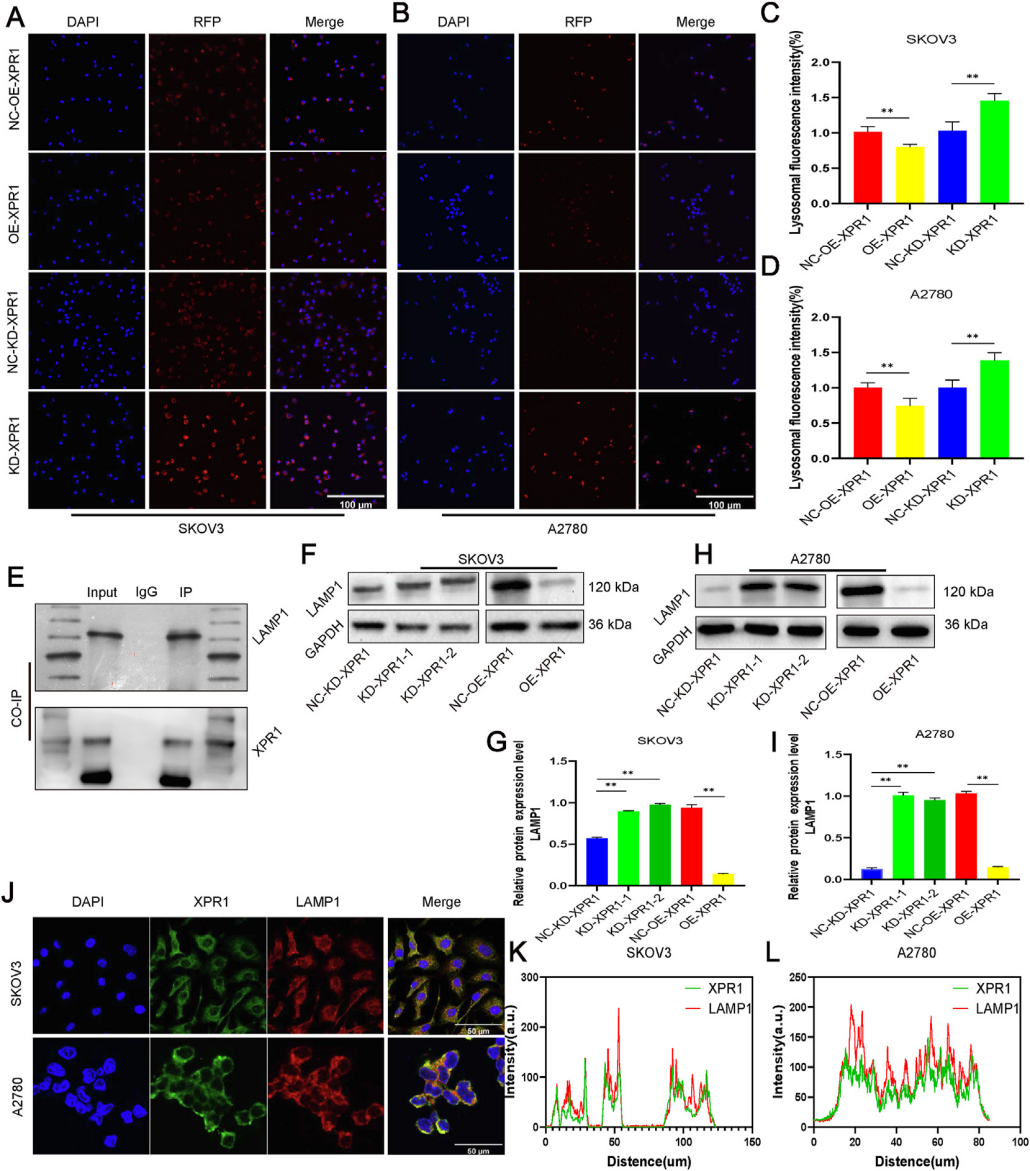
XPR1 interacted with LAMP1 and regulated its expression. **(A)** SKOV3 and A2780 cells were transfected with KD-XPR1, NC-KD-XPR1, OE-XPR1, and NC-OE-XPR1. The lysosome probe (red) was used to detect lysosome morphology 48 h after transfection. Scale bar: 100 μm. Image-Pro Plus 6.0 was used for fluorescence intensity analysis. **(B)** The co-immunoprecipitation assay was used to detect LAMP1 and XPR1 interactions in SKOV3 cells. **(C)** The co-localization of LAMP1 (red) and XPR1 (green) was detected by immunofluorescence double labeling in A2780 and SKOV3 cells. Scale bar: 50 μm. **(D)** SKOV3 and A2780 cells were transfected with KD-XPR1, NC-KD-XPR1, OE-XPR1, and NC-OE-XPR1. LAMP1 protein was detected by Western blot 48 h after transfection. Each experiment was repeated three times. Error bars represent standard deviation. ∗∗*P* < 0.01.

Lysosomes participate in the formation of autophagolysosomes. So, we used lysosome probes to detect the influence of XPR1 on lysosomes. Lysosome quantity rose following XPR1 inhibition in SKOV3 and A2780 ovarian cancer cells, contrasting with a decline following XPR1 up-regulation. So, we hypothesized that XPR1 was involved in lysosomal function (Fig. 5A–D). Biogrid reported that LAMP1/2/3 may be the binding partners of XPR1 (https://thebiogrid.org/). We confirmed by co-immunoprecipitation that XPR1 and LAMP1 proteins bound directly (Fig. 5E). Additionally, it was discovered that the levels of LAMP1 protein rose following XPR1 suppression, but decreased with XPR1 up-regulation in SKOV3 and A2780 ovarian cancer cells (Fig. 5F–I). Simultaneously, XPR1 and LAMP1 proteins were found to be in the same location in the cytoplasm of SKOV3 and A2780 cells from ovarian cancer (Fig. 5J–L).

### XPR1 controlled the PI3K/Akt/mTOR signaling cascade

**Figure 6:**
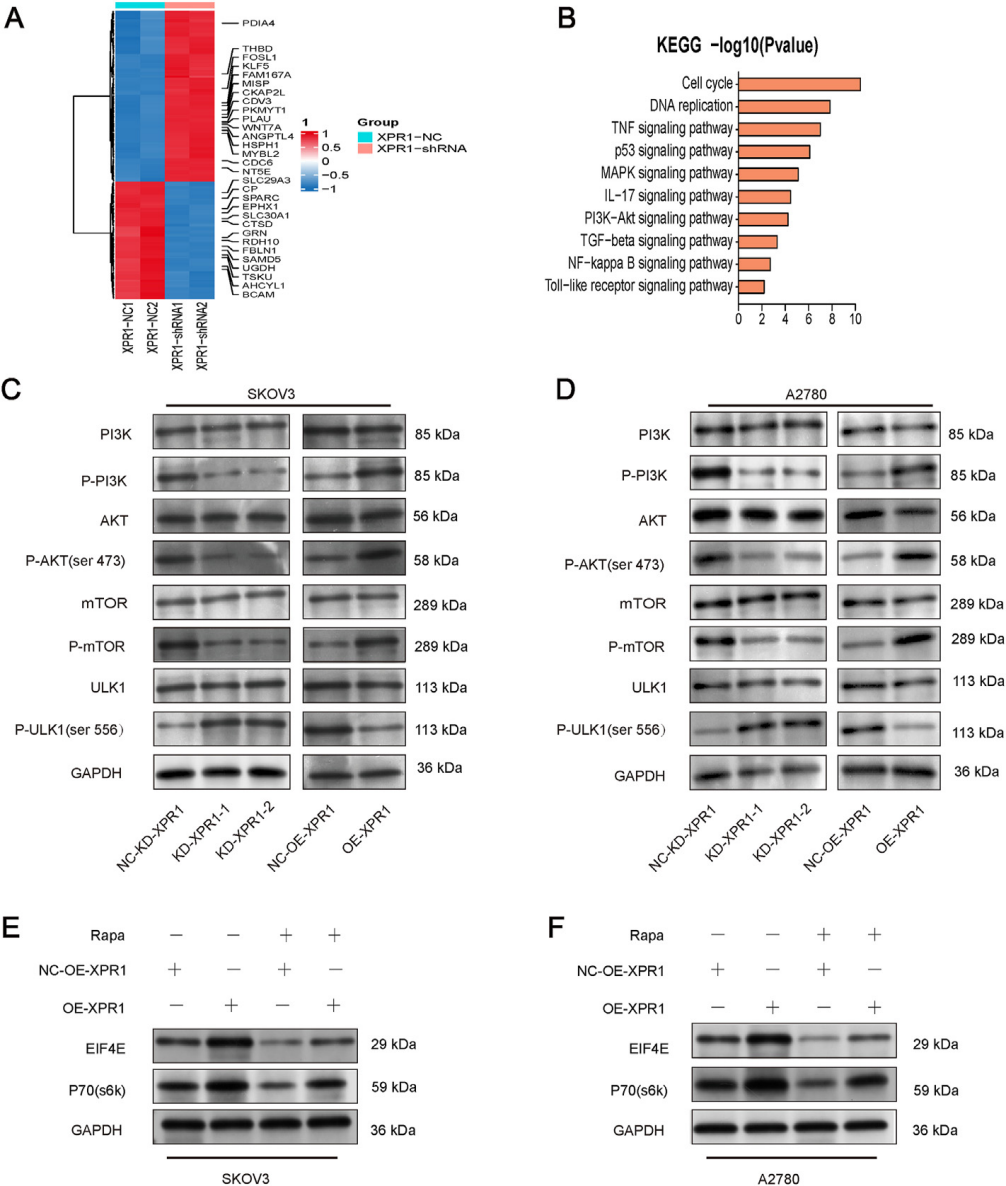
XPR1 regulated the PI3K/Akt/mTOR signaling pathway. SKOV3 cells were transfected with XPR1 (XPR1-975) and control siRNAs. The RNA was collected 48 h after transfection. Transcriptomically differentially expressed genes were detected by next-generation sequencing technology. **(A)** Heat maps of the top 100 differentially expressed mRNAs. **(B)** KEGG analyzed the signaling pathways of differentially expressed genes. **(C)** XPR1-975 siRNA (named XPR1-1), XPR1-1860 siRNA (named XPR1-2), and control siRNA (named NC-KD-XPR1) were transfected into SKOV3 and A2780 cells. XPR1-overexpressing lentiviral vectors (named OE-XPR1) or control lentiviral vectors (named NC-OE-XPR1) were transfected into SKOV3 and A2780 cells. The protein was collected and the expression levels of PI3K, p-PI3K, Akt, p-Akt, mTOR, p-mTOR, ULK1, and p-ULK1 proteins were detected by Western blot 48 h after transfection. **(D)** XPR1-overexpressing lentiviral vectors (named OE-XPR1) or control lentiviral vectors (named NC-OE-XPR1) were transfected into SKOV3 and A2780 cells. The mTOR signaling pathway inhibitor rapamycin was added 48 h after transfection. The protein was collected and the expression level of EIF4E and p70 protein was detected by Western blot after another 48 h.

We used next-generation sequencing to detect the transcriptomic change pattern of ovarian cancer SKOV3 cells after silencing XPR1 to further investigate the mechanism of XPR1 regulation of autophagy. After silencing XPR1, it was discovered that 432 genes showed a significant increase in expression, while 562 genes displayed a significant decrease in expression (Fig. 6A). The analysis of KEGG signaling pathways indicated that the differentially expressed genes were associated with MAPK and PI3K-Akt signaling pathways (Fig. 6B). Silencing XPR1 in SKOV3 and A2780 cells led to reduced levels of p-Akt, P-PI3K, and p-mTOR, with an increase in P-ULK expression. Conversely, XPR1 overexpression showed an opposite pattern on the expression of these proteins (Fig. 6C, D; Fig. S2A–H). The overexpression of XPR1 increased the expression of EIF4E and P70. The effect of XPR1 on EIF4E and P70 was inhibited by adding mTOR pathway inhibitor rapamycin (Fig. 6E, F; Fig. S2I–L). The data suggested that XPR1 controlled the PI3K/Akt/mTOR signaling pathway.

### Autophagy mediated by XPR1 regulated the expression of MHC-1

**Figure 7:**
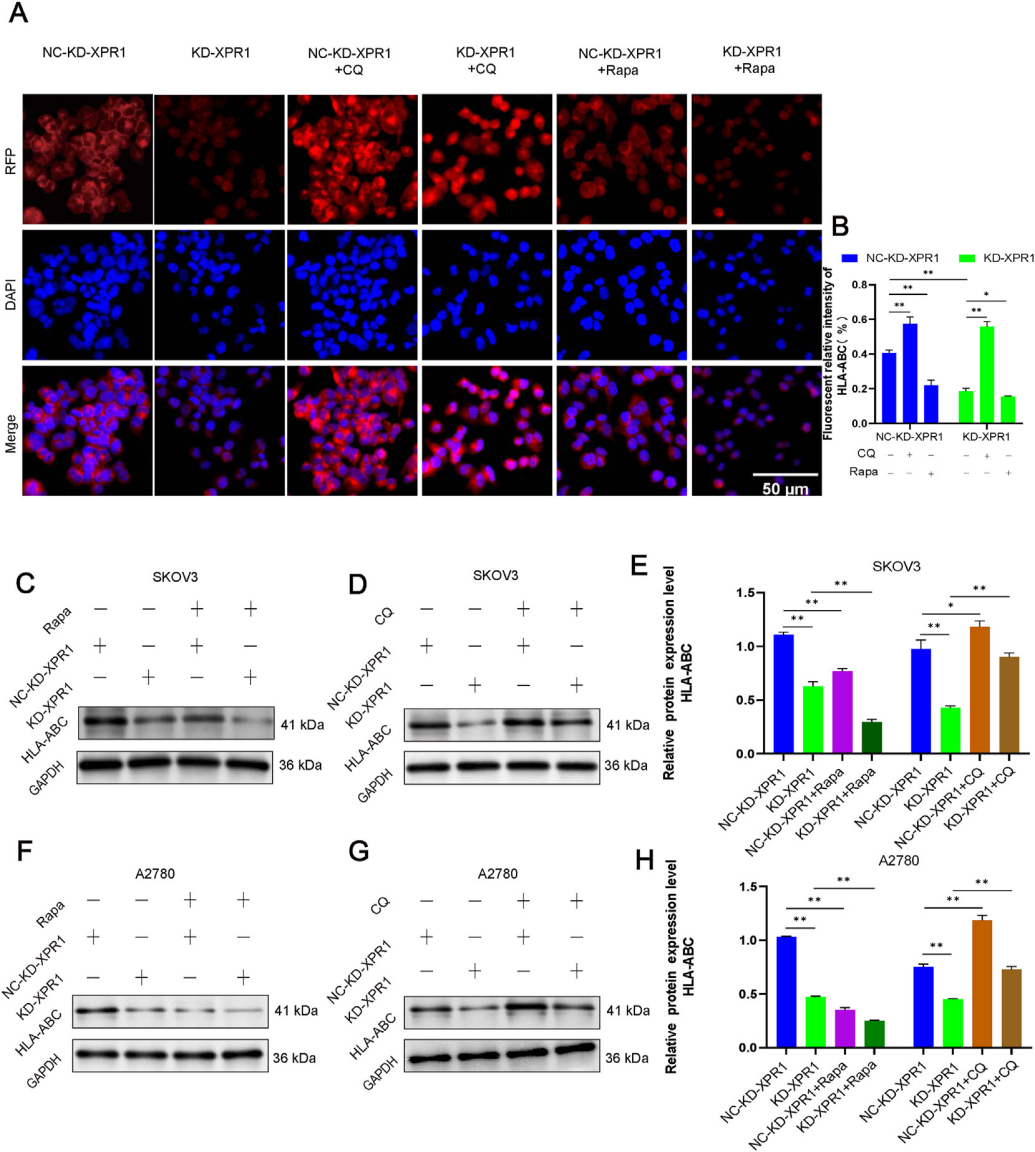
Autophagy mediated by XPR1 regulated the expression of MHC-1. XPR1-975 siRNA (named KD-XPR1) and control siRNA (named NC-KD-XPR1) were transfected into SKOV3 and A2780 cells. XPR1-overexpressing lentiviral vectors (named OE-XPR1) or control lentiviral vectors (named NC-OE-XPR1) were transfected into SKOV3 and A2780 cells. The cells were simultaneously transfected with LAMP1 siRNA or LAMP1 overexpressing lentiviral vector. The protein was collected, and the protein expression level of LC3-II, P62, and HLA-ABC was detected by Western blot 48 h after transfection. Each experiment was repeated three times. Error bars represent standard deviation. ∗∗*P* < 0.01.

**Figure 8:**
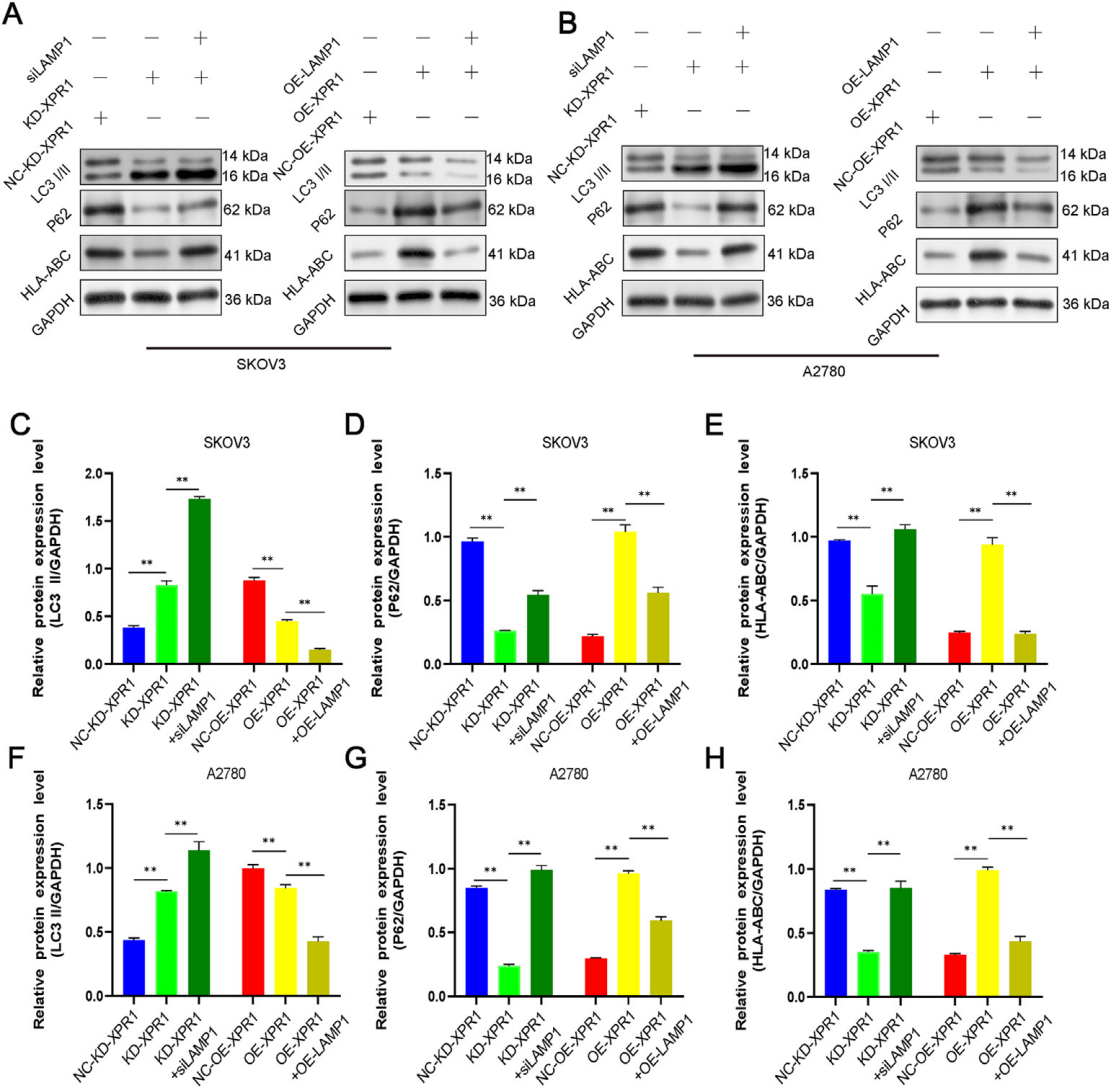
Autophagy inhibitors increased the expression of MHC-I molecules. **(A)** XPR1-975 siRNA (named KD-XPR1) and control siRNA (named NC-KD-XPR1) were transfected into SKOV3 cells. XPR1-overexpressing lentiviral vectors (named OE-XPR1) or control lentiviral vectors (named NC-OE-XPR1) were transfected into SKOV3 and A2780 cells. Autophagy inhibitor chloroquine and autophagy activator rapamycin were added to the control and XPR1 gene silencing groups, respectively. The localization of MHC-1 (red) in SKOV3 cells was detected by immunofluorescence. Original magnification: 200 × . Scale bar: 50 μm. **(B, C)** XPR1-975 siRNA (named KD-XPR1) and control siRNA (named NC-KD-XPR1) were transfected into SKOV3 cells. XPR1-overexpressing lentiviral vectors (named OE-XPR1) or control lentiviral vectors (named NC-OE-XPR1) were transfected into SKOV3 and A2780 cells. Autophagy inhibitor chloroquine and autophagy activator rapamycin were added to the control and XPR1 gene silencing groups, respectively. Western blot was used to detect the expression of MHC-I protein. Each experiment was repeated three times. Error bars represent standard deviation. ∗*P* < 0.05, ∗∗*P* < 0.01.

MHC-I presence on the surface of ovarian cancer cells plays a role in the immune response against tumors by CD8^+^ T cells. Consequently, we investigated how XPR1 impacts the expression of MHC-I in SKOV3 ovarian cancer cells. After silencing XPR1, we noticed an increase in the ratio of LC3-II/LC3-I and a decrease in the expression of P62 and MHC-1. Additionally, the ratio of LC3-II to LC3-I decreased, while the expression of P62 and MHC-1 increased following the overexpression of XPR1 (Fig. 7A–H). Additionally, it was discovered that the levels of MHC-I on the surface of A2780 ovarian cancer cells decreased when treated with the autophagy stimulant rapamycin, in contrast to the control group. Silencing XPR1 led to a notable decrease in the presence of MHC-I on the cell surface. MHC-I expression on the cell membrane rose following the introduction of the autophagy inhibitor chloroquine (Fig. 8A, B). This indicated that blocking autophagy led to higher levels of MHC-I molecules being produced. Western blot results further confirmed that the regulation of MHC-I molecules by XPR1 depended on LAMP1 and autophagy (Fig. 8C–H).

### XPR1 promoted ovarian cancer cell growth in nude mice

**Figure 9:**
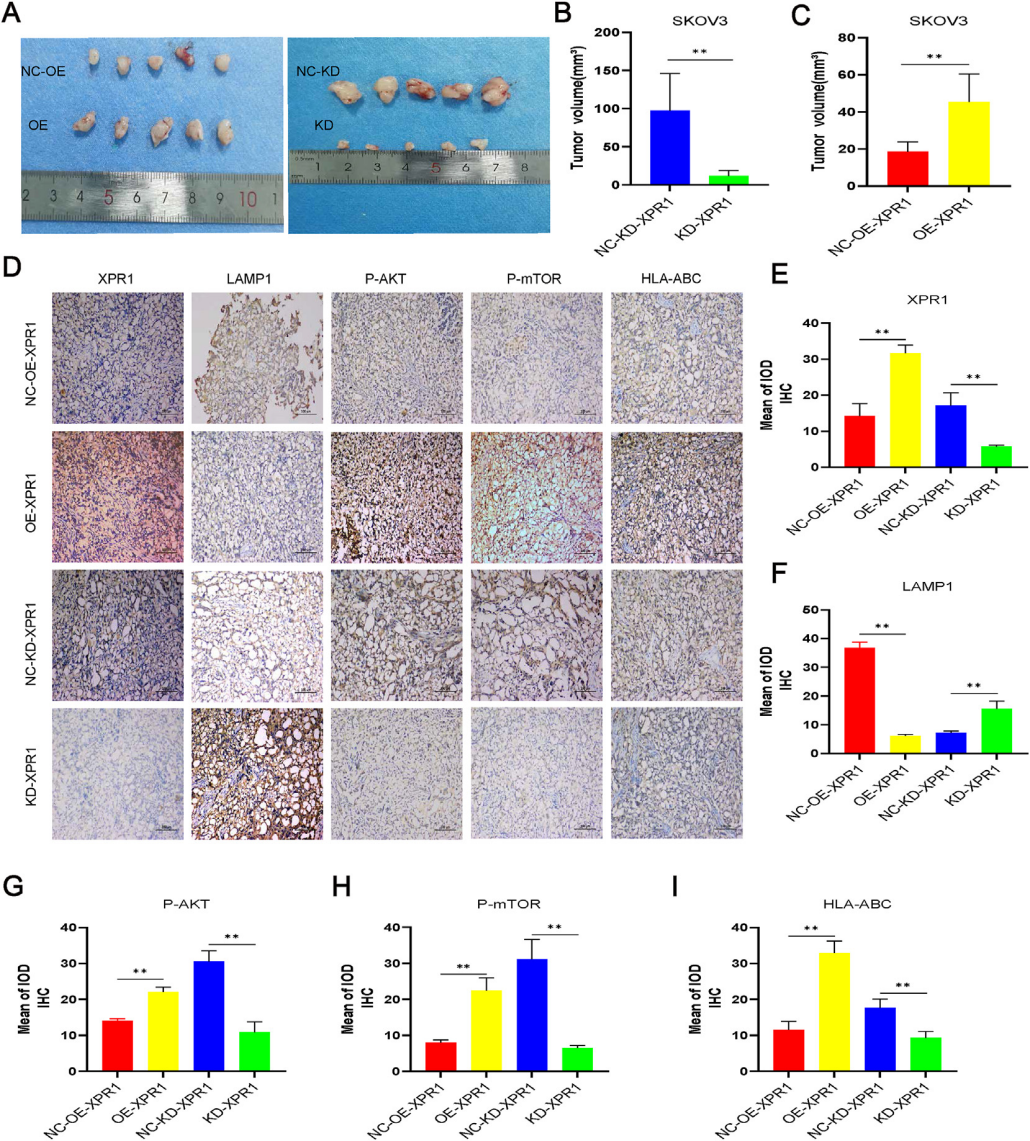
XPR1 regulated tumorigenesis in a nude mouse model. **(A)** Mean tumor volume on day 28 after SKOV3 cell injection. SKOV3 cells transfected with lentiviral vector XPR1-975 shRNA (named KD) and control vector (named NC-KD), XPR1-overexpressing lentiviral vector (named OE), or control lentiviral vector (named NC-OE) cells were implanted subcutaneously into the left armpit. **(B)** Immunohistochemical analysis of XPR1, LAMP1, p-Akt, p-mTOR, and MHC-I expression was performed on tumor xenografts. Representative images are shown. Original magnification: 200 × . Scale bar: 100 μm. Error bars represent standard deviation. ∗*P* < 0.05, ∗∗*P* < 0.01.

In nude mice, the growth of ovarian cancer SKOV3 cells was enhanced by the overexpression of XPR1 (Fig. 9A–C). Silencing XPR1 inhibited its growth. Overexpression of XPR1 led to an increase in the levels of p-Akt, p-mTOR, and MHC-I while causing a decrease in the expression of LAMP1. The opposite expression pattern was observed after silencing XPR1 (Fig. 9D–I).

### XPR1 promoted ovarian cancer cell growth in C57 mice

**Figure 10:**
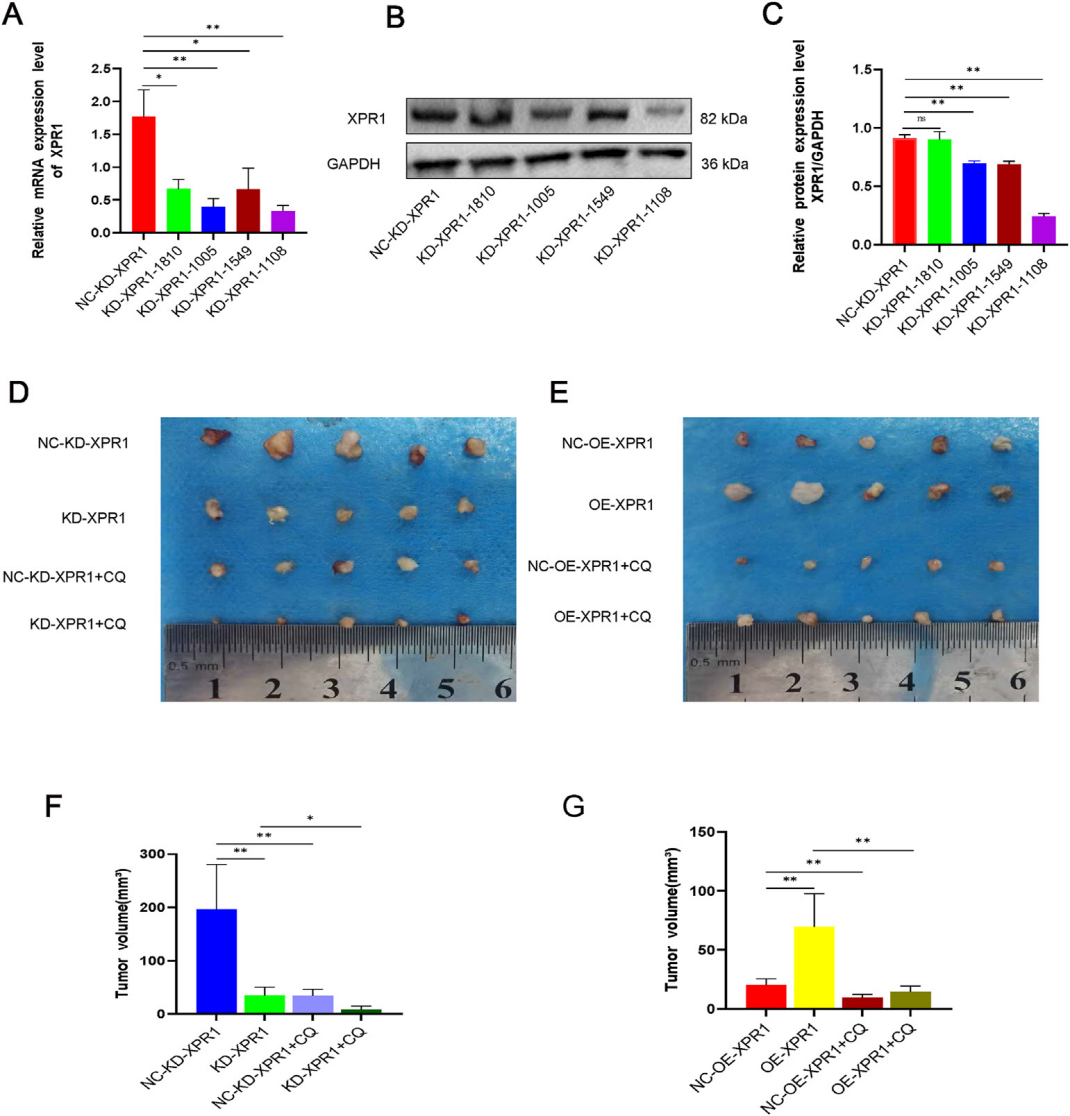
XPR1 promoted ovarian cancer cell growth in C57 mice. **(A, B)** XPR1 siRNA was transfected into ID8 cells. After 48 h, mRNA was extracted, and quantitative PCR and Western blot were used to detect the mRNA and protein expression of XPR1, respectively. **(C)** Mean tumor volume on day 28 after ID8 cell injection. ID8 cells, transfected with lentiviral vectors carrying XPR1-1108 shRNA (named KD), and those with control vectors (named NC-KD), XPR1-overexpressing lentiviral vector (named OE), or control lentiviral vector (named NC-OE) cells were subcutaneously implanted into the left armpit. ∗*P* < 0.05, ∗∗*P* < 0.01.

**Figure 11:**
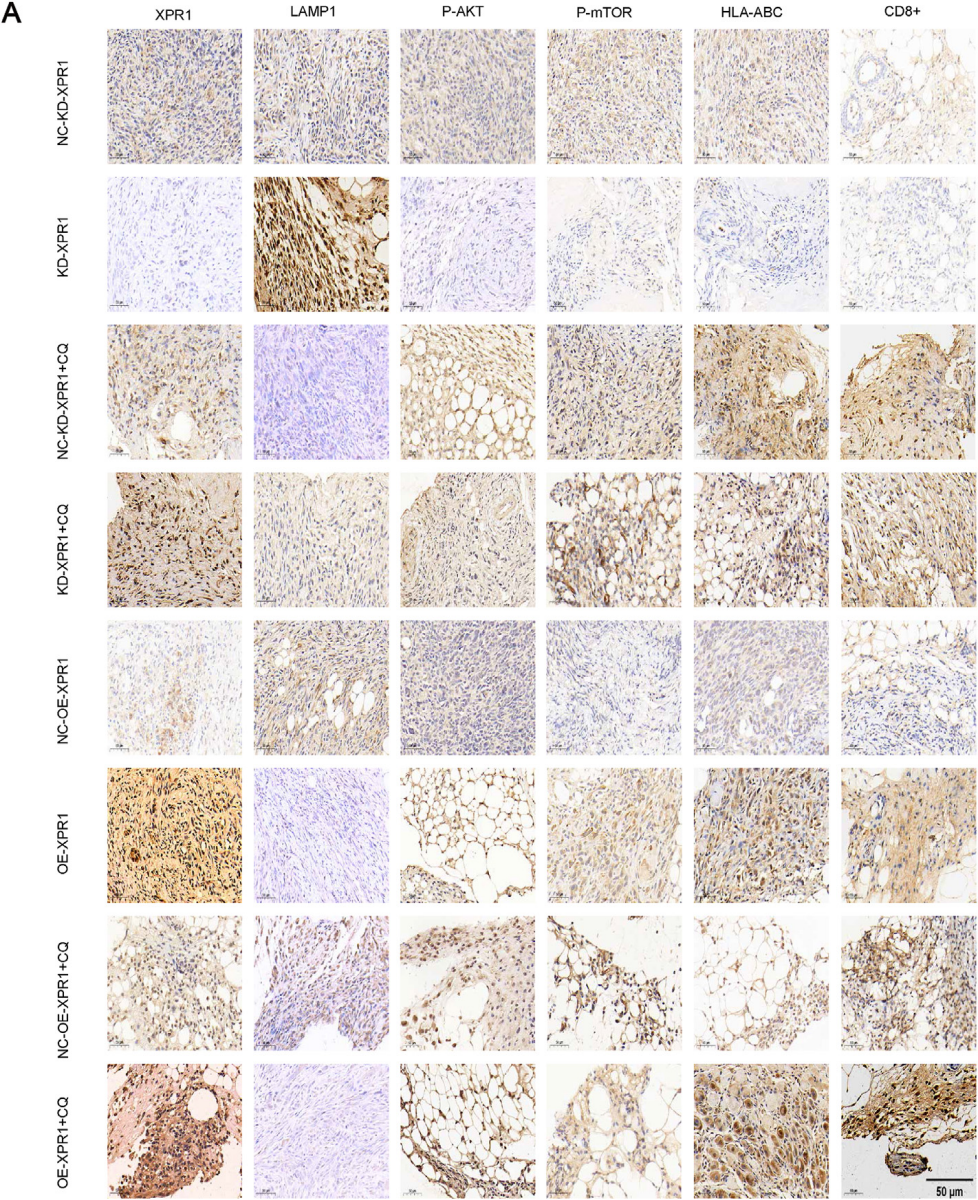
Protein expression in the C57 mice ovarian cancer model was detected by immunohistochemistry. Immunohistochemical analysis of XPR1, LAMP1, p-Akt, p-mTOR CD8, and MHC-I expression was performed on tumor xenografts. Representative images are shown. Original magnification: 200 × . Scale bar: 50 μm.

XPR1 mRNA and protein levels decreased after XPR1 silencing in ID8 cells (Fig. 10A–C). In C57 mice, the growth of ovarian cancer ID8 cells was enhanced by the overexpression of XPR1. Silencing XPR1 combined with chloroquine significantly inhibited the growth of tumor lesions (Fig. 10D–G). Following XPR1 overexpression, there was an increase in the levels of p-Akt, p-mTOR, MHC-I, and CD8, while the expression of LMAP1 decreased. Chloroquine was included to enhance the production of CD8 and MHC-I proteins. The opposite expression pattern appeared after silencing XPR1 (Fig. 11A; Fig. S3A–F).

## Discussion

This study found that XPR1 expression was up-regulated in ovarian cancer tissues, and its high expression was related to the ovarian cancer stage, overall survival, and progression-free survival. The higher the stage of XPR1 in ovarian cancer tissue, the higher its expression. Its high expression suggests a poor prognosis. Additional research revealed that XPR1 suppressed autophagy flux by interacting with LAMP1 and the PI3K/Akt/mTOR pathway. XPR1 regulated the localization and expression of MHC-I molecules in the ovarian cancer cell membrane through autophagy. Silencing XPR1 combined with the treatment with autophagy inhibitor chloroquine significantly inhibited tumor growth in mouse ovarian cancer models. To our knowledge, we were the first to discover that XPR1 can regulate autophagy and tumor immunity and that XPR1 regulates tumor immunity through the lysosomal protein LAMP1. The findings indicated that XPR1 could serve as a promising target for diagnosing and treating ovarian cancer.

XPR1 was pinpointed as a gene linked to the progression of ovarian clear cell carcinoma through the utilization of the CRISPR/Cas9 system. Also, silencing XPR1 could induce the apoptosis of ovarian cancer cells.^22^ Using the CRISPR-Cas9 loss-of-function strategy, XPR1 was found to promote ovarian cancer growth. Knocking out XPR1 caused phosphate accumulation in cells, which, in turn, led to ovarian cancer cell death. Therefore, XPR1 could be a potential therapeutic target for ovarian cancer.^23^ In this study, we used the CRISPR/Cas9 system to identify XPR1 as a potential regulator of autophagy. This suggested the involvement of XPR1 in ovarian cancer growth and autophagy. We also found that XPR1 expression increased in ovarian cancer, and its high expression indicated ovarian cancer staging and a worse prognosis. This implies that XPR1 may serve as a marker for diagnosing and predicting the outcome of ovarian cancer.

The involvement of autophagy in ovarian cancer remains a topic of debate. Certain research indicated that autophagy supported the development of ovarian cancer, whereas other studies suggested it hindered the progression of ovarian cancer.^24^, ^25^, ^26^ In this study, we confirmed through a series of experiments that XPR1 inhibited autophagy flux. Additionally, it was discovered that XPR1 enhanced the proliferation of ovarian carcinoma. Several research studies have verified that the initiation of the PI3K/Akt/mTOR signaling cascade suppresses autophagy in ovarian cancer. The research showed that XPR1 triggered the PI3K/Akt/mTOR pathway to control autophagy. LAMP1, as a membrane protein of lysosomes, regulates the morphology and function of lysosomes. At the same time, LAMP1 participates in the formation of autophagolysosomes and promotes autophagy.^27^^,^^28^ Our research revealed that XPR1 suppressed the formation of lysosomes. Further studies showed that XPR1 and LAMP1 were colocalized in the cytoplasm and directly interacted with each other. XPR1 could regulate the expression of LAMP1. Therefore, XPR1 could regulate autophagy by influencing lysosomes through LAMP1.

Certain research has indicated that autophagy can break down the presence of MHC-I on the surface of cancer cells, allowing them to evade detection by T-cells. This was one reason for the poor efficacy of PD-1 and CTLA-4 checkpoint inhibitors. The knockout of autophagy-related genes RB1CC1, ATG9A, and ATG12 significantly increased tumor cell susceptibility to T-cell killing in breast and colon cancers and enhanced the anti-cancer effect of PD-1 and CTLA-4 checkpoint inhibitors.^4^ The absence of MHC-I antigens in the ID8 ovarian cancer cell line indicated a connection between immune evasion in ovarian cancer and MHC-I molecules.^29^ Ovarian cancer cell lines OVCAR8 and CAOV3 exhibited reduced levels of MHC-I antigen expression, leading to their resistance against T-cell cytotoxicity.^30^ This research revealed that chloroquine, an inhibitor of autophagy, enhanced the presence of MHC-I on the surfaces of ovarian cancer cells. Rapamycin, an autophagy inducer, decreased MHC-I expression in ovarian cancer cells. Simultaneously, it was discovered that XPR1 enhanced the production of MHC-I molecules in ovarian cancer cells. Inhibiting XPR1 along with chloroquine led to a significant decrease in the growth of ovarian cancer cells in a live organism. This suggested that the inhibition of autophagy might enhance the efficacy of immunotherapy in ovarian cancer. Therefore, XPR1 could serve as a promising target for treating ovarian cancer.

In conclusion, our research revealed that XPR1 controlled the MHC-I expression in ovarian cancer cells by utilizing the autophagy pathway. Additionally, we discovered that the inhibition of ovarian cancer lesion growth was achieved by combining targeted XPR1 with autophagy inhibitors. The research we conducted established a theoretical foundation for utilizing autophagy inhibitors in conjunction with immune checkpoint inhibitors in clinical settings.

Limitations still exist in this study. First, this study used two cell lines, SKOV3 and A2780, which cannot fully reflect all epithelial ovarian cancers. Second, XPR1 down-regulation used gene silencing technology, and did not use gene editing technology; third, the animal experiment used nude mouse subcutaneous tumor formation technology and did not use ovarian cancer *in situ* tumor technology, which cannot reflect the abdominal metastasis process of ovarian cancer. Finally, the role of XPR1 in the occurrence of ovarian cancer needs to be confirmed using XPR1 gene knockout and transgenic mice. In our subsequent studies, we will use the above technologies to explain the function and mechanism of XPR1's action in ovarian cancer.

In this study, we discovered the relationship between autophagy and tumor immunity. Inhibiting autophagy can promote the expression of MHC-I molecules. Since ovarian cancer immunotherapy is less effective, autophagy inhibitors combined with PD-1 antibodies may increase the efficacy of tumor immunotherapy. In the future, we plan to continue to combine basic and clinical experiments to further study the relationship between autophagy and ovarian cancer immunity. Provide a theoretical basis for the use of autophagy inhibitors in the field of tumor immunotherapy.

## Funding

This work was supported by grants from the Kuanren Talents Program of the Second Affiliated Hospital of Chongqing Medical University; Senior Medical Talents Program of Chongqing for Young and Middle-aged; Chongqing Science and Technology Bureau Project (CSTB2024TIAD-KPX0038).

## CRediT authorship contribution statement

**Hui Wang:** Resources. **Xiaodong Luo:** Formal analysis. **Bo Yang:** Resources. **Furong Tang:** Conceptualization. **Xingwei Jiang:** Conceptualization. **Hongtao Zhu:** Conceptualization. **Jianguo Hu:** Writing – original draft, Project administration, Methodology.

## Conflict of interests

The authors affirm that they possess no conflicting concerns.
