# Supplementary figures and images for: XPR1 promotes ovarian cancer growth and regulates MHC-I through autophagy
Source: Genes Dis. 2024 Dec 27;12(5):101507. doi: 10.1016/j.gendis.2024.101507 (PMC12242406; doi:10.1016/j.gendis.2024.101507)

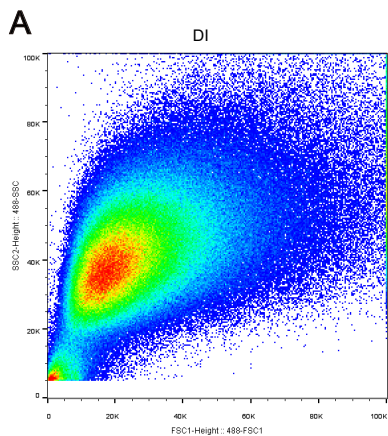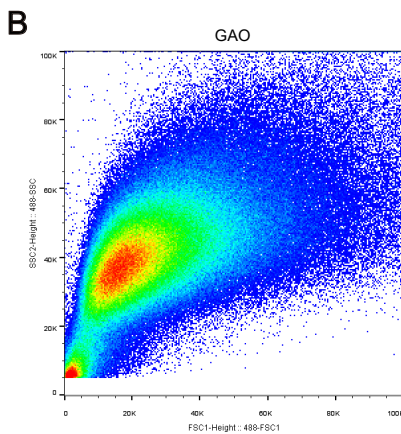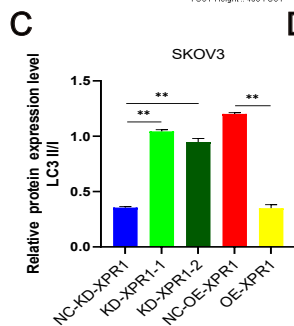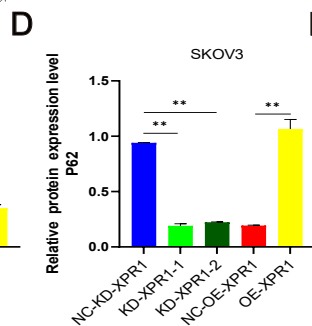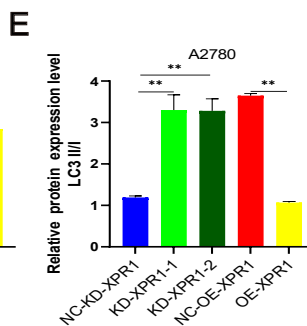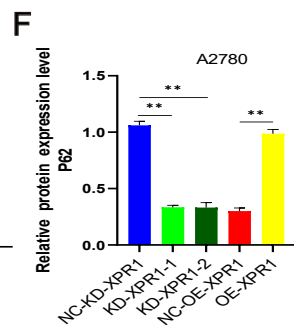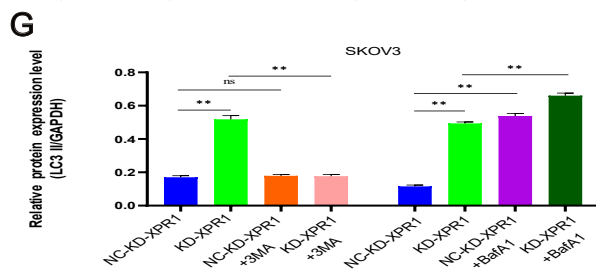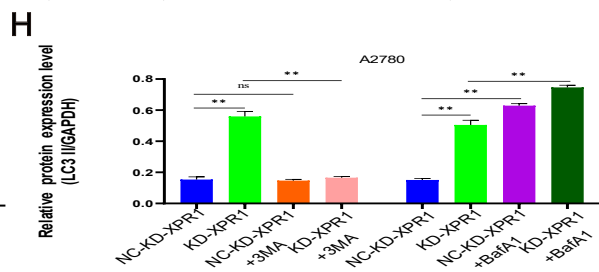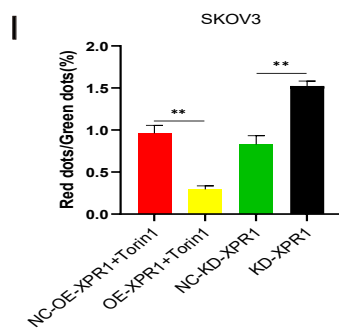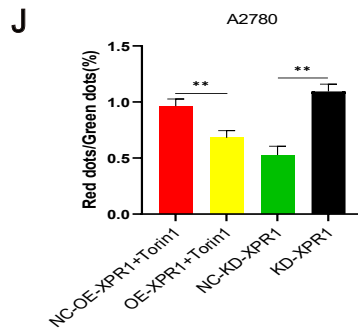

Supplement: Multimedia component 1 [file mmc1.pdf]

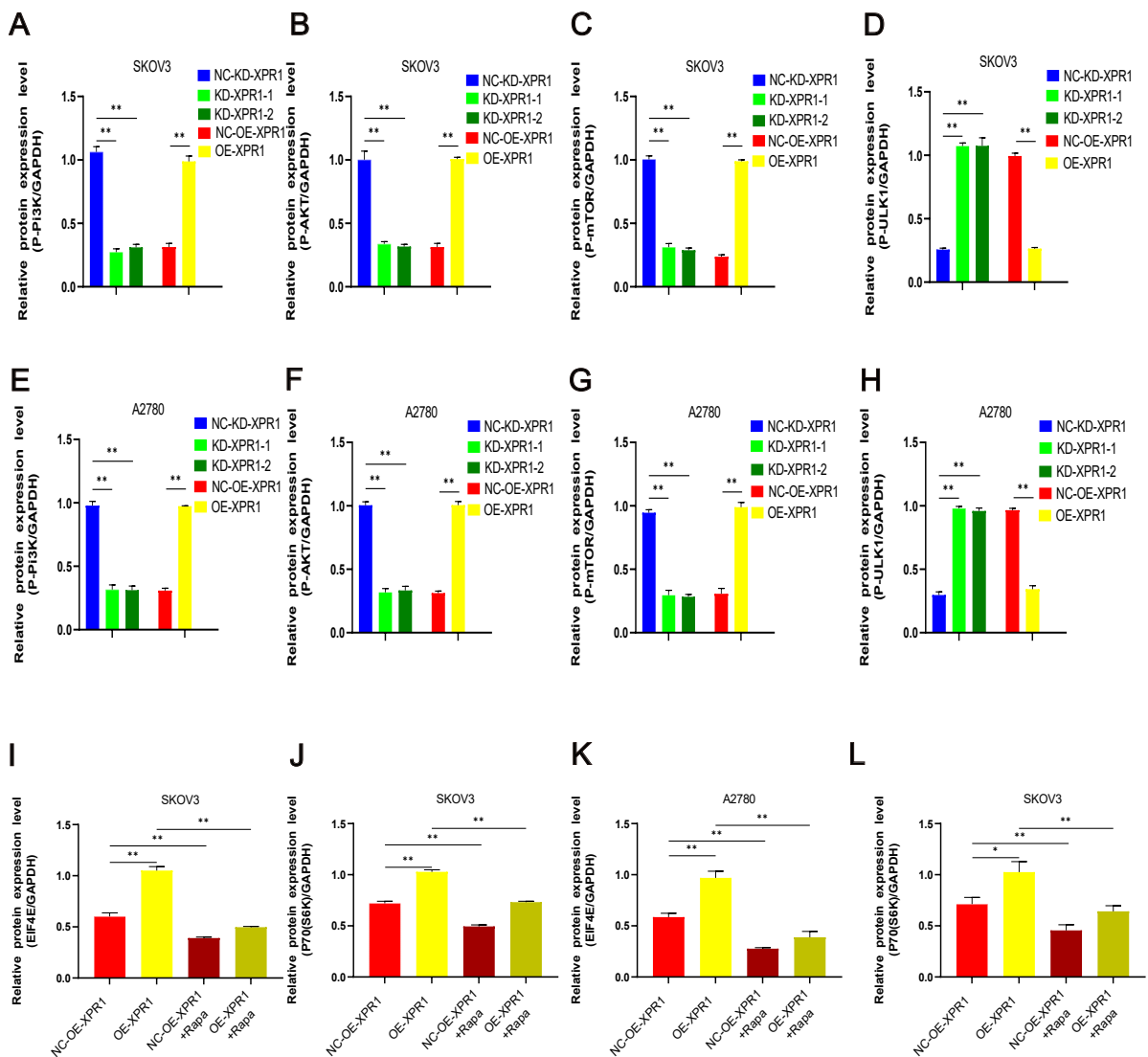

Supplement: Multimedia component 2 [file mmc2.pdf]

**A**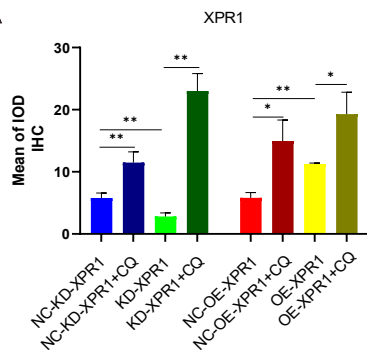**B**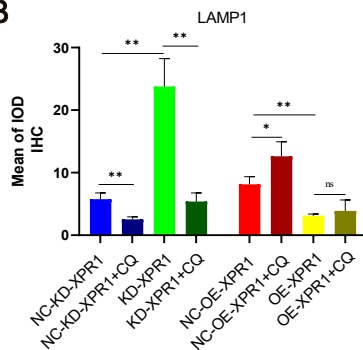**C**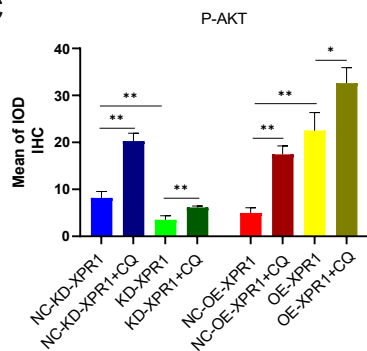**D**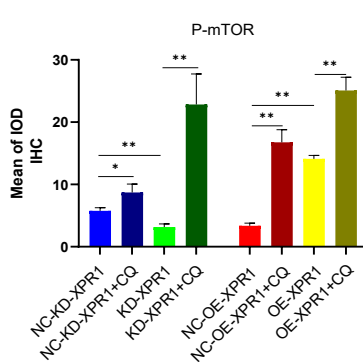**E**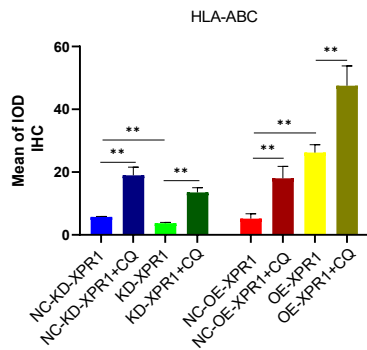**F**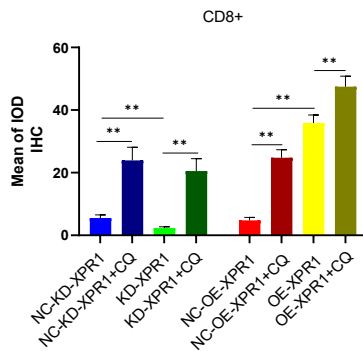

Supplement: Multimedia component 3 [file mmc3.pdf]
